# Sustained Protection in Mice Immunized with Fractional Doses of *Salmonella* Enteritidis Core and O Polysaccharide-Flagellin Glycoconjugates

**DOI:** 10.1371/journal.pone.0064680

**Published:** 2013-05-31

**Authors:** Raphael Simon, Jin Y. Wang, Mary A. Boyd, Mohan E. Tulapurkar, Girish Ramachandran, Sharon M. Tennant, Marcela Pasetti, James E. Galen, Myron M. Levine

**Affiliations:** 1 Center for Vaccine Development, University of Maryland School of Medicine, Baltimore, Maryland, United States of America; 2 Department of Medicine, University of Maryland School of Medicine, Baltimore, Maryland, United States of America; 3 Department of Pediatrics, University of Maryland School of Medicine, Baltimore, Maryland, United States of America; 4 Division of Pulmonary and Critical Care, University of Maryland School of Medicine, Baltimore, Maryland, United States of America; Indian Institute of Science, India

## Abstract

Non-typhoidal *Salmonella* (NTS) serovars *S*. Enteritidis and *S*. Typhimurium are a major cause of invasive bacterial disease (e.g., bacteremia, meningitis) in infants and young children in sub-Saharan Africa and also occasionally cause invasive disease in highly susceptible hosts (young infants, the elderly, and immunocompromised subjects) in industrialized countries. No licensed vaccines exist against human NTS infections. NTS core and O polysaccharide (COPS) and FliC (Phase 1 flagellin subunits) each constitute protective antigens in murine models. *S*. Enteritidis COPS conjugated to FliC represents a promising vaccine approach that elicits binding and opsonophagocytic antibodies and protects mice against lethal challenge with virulent *S*. Enteritidis. We examined the protective efficacy of fractional dosages of *S*. Enteritidis COPS:FliC conjugate vaccines in mice, and also established that protection can be passively transferred to naïve mice by administering sera from mice immunized with conjugate. Mice were immunized with three doses of either 10 µg, 2.5 µg (full dose), 0.25 µg, or 0.025 µg *S*. Enteritidis COPS:FliC conjugate at 28 day intervals. Antibody titers to COPS and FliC measured by ELISA fell consonant with progressively smaller vaccine dosage levels; anti-FliC IgG responses remained robust at fractional dosages for which anti-COPS serum IgG titers were decreased. Nevertheless, >90% protection against intraperitoneal challenge was observed in mice immunized with fractional dosages of conjugate that elicited diminished titers to both FliC and COPS. Passive transfer of immune sera from mice immunized with the highest dose of COPS:FliC to naïve mice was also protective, demonstrating the role of antibodies in mediating protection. These results provide important insights regarding the potency of *Salmonella* glycoconjugate vaccines that use flagellin as a carrier protein.

## Introduction

Non-typhoidal *Salmonella* (NTS) infections are a global problem, with distinct regional clinico-epidemiological differences. In industrialized countries, NTS are common causes of bacterial gastroenteritis and occasionally cause invasive disease (meningitis, septicemia, bacteremia, etc.) in susceptible groups such as young infants, the elderly and immunocompromised subjects [1]. In sub-Saharan Africa, invasive salmonellosis caused by multiple antibiotic-resistant NTS strains are among the most common causes of invasive bacterial disease in infants and young children, with a case fatality rate between 15–30% [2]. Importantly, two serovars, *S*. Typhimurium (and monophasic variants) and *S*. Enteritidis, cause 80–95% of invasive disease in sub-Saharan Africa [1], [2], making the concept of control by vaccination epidemiologically feasible.

*Salmonella* lipopolysaccharide (LPS) and flagellin (the structural protein subunit of polymeric flagella filaments) are protective antigens in animal models [3], [4]. The conserved core and serogroup-specific O polysaccharide (COPS) constitute the polysaccharide portion of LPS. Unconjugated NTS COPS is a poor immunogen that does not elicit immunologic memory in animal models [4], [5] and unconjugated bacterial polysaccharides, including capsular polysaccharides, are also, in general, weakly immunogenic in human infants [6]. In contrast, conjugation of *Salmonella* COPS with proteins has been shown to improve anti-polysaccharide humoral responses and to induce protection in mice [4], [5], [7].

We reported previously that *S*. Enteritidis COPS:FliC conjugates were immunogenic and protective in mice against virulent *S*. Enteritidis strain R11 (originally isolated from the blood of a Malian child) and the antibodies in post-vaccination sera manifested opsonophagocytic activity [4]. We report herein that *S*. Enteritidis COPS:FliC conjugates protect even when administered in fractional dosages that elicit diminished anti-FliC and COPS antibody responses (compared to the 2.5 µg “full dose” that was reported previously [4]), and that passive transfer of serum from conjugate-immunized mice protects naïve mice against otherwise lethal *S*. Enteritidis challenge.

## Materials and Methods

### Ethics Statement

All animal experiments carried out in this work were approved by the University of Maryland Baltimore Office of Animal Welfare Assurance (OAWA), under approved Animal Use Protocol 0909007.

### Bacterial Strains

Characteristics and growth conditions of wild-type *S.* Enteritidis R11 and attenuated derivative CVD 1941 (Δ*guaBA* Δ*clpP*) have been previously described [4].

### Purification of LPS, COPS and Flagellin, and Synthesis of COPS:FliC Conjugates

Purification of LPS, COPS and FliC from CVD 1941 and their characterization were performed as described [4]. Direct conjugation between COPS and FliC monomers was accomplished at a 1∶1 ratio of polysaccharide to protein, using 1-cyano-4-dimethylaminopyridinium tetrafluoroborate (CDAP, Research Organics, OH) [8]. Unreacted protein and polysaccharide were removed by size-exclusion chromatography with Superdex 200 (GE/Amersham, NJ) and anion exchange Q membrane chromatography (Sartorius, Germany) [4].

### Mice

Female outbred CD-1 mice (8–10 week old) were purchased from Charles River Laboratories (Wilmington, MA). Animal protocols were approved by the University of Maryland School of Medicine Institutional Animal Care and Use Committee.

### Immunization and Challenge

Mice were injected intramuscularly (IM) in the right hind limb at 0, 28 and 56 days with either 10 µg (a 4-fold dose), 2.5 µg (full dose), 0.25 µg (1/10^th^ dose) or 0.025 µg (1/100^th^ dose) of polysaccharide conjugated to FliC in 50 µl of sterile PBS. Sera were obtained before vaccination and at day 77. Mice were challenged intraperitoneally (IP) on day 84, with 1×10^6^ CFU of *S*. Enteritidis R11 (IP LD_50_ = 2.2×10^5^). To assess the protective effect of passively transferred antibodies, naïve mice were injected intravenously (IV) through the tail vein with 100 µl of PBS (negative controls) or with pooled sera from mice immunized with PBS (normal serum negative controls) or with 10 µg of COPS:FliC (immune serum) diluted with PBS to 434 ELISA Units (EU) of anti-LPS IgG and 500,000 EU anti-FliC IgG per dose. Mice were infected IP 2–3 hours later with 5×10^5^ CFU of *S*. Enteritidis R11. Mice were monitored for 21 days after challenge, recording overall health, weight loss and mortality. Moribund mice exhibiting signs including lethargy, non-responsiveness and ≥20% weight loss were euthanized and recorded as dead.

### Serum Antibody Analysis

IgG levels against LPS or FliC were measured by ELISA, and end-point titers reported as ELISA units (EU)/ml, as previously described [4]. Seropositivity was defined as a titer four-fold above the Geometric Mean Titer (GMT) of sera from mice immunized with PBS.

### Statistical Analysis

Serological responses of groups of mice were compared by Mann-Whitney rank sum test and mortality incidences by Fisher’s exact test (FET), using Sigma-Stat software package; p≤0.05 was considered significant.

## Results

### Humoral Response to Full and Fractional Dosages of COPS:FliC Conjugate

**Figure 1:**
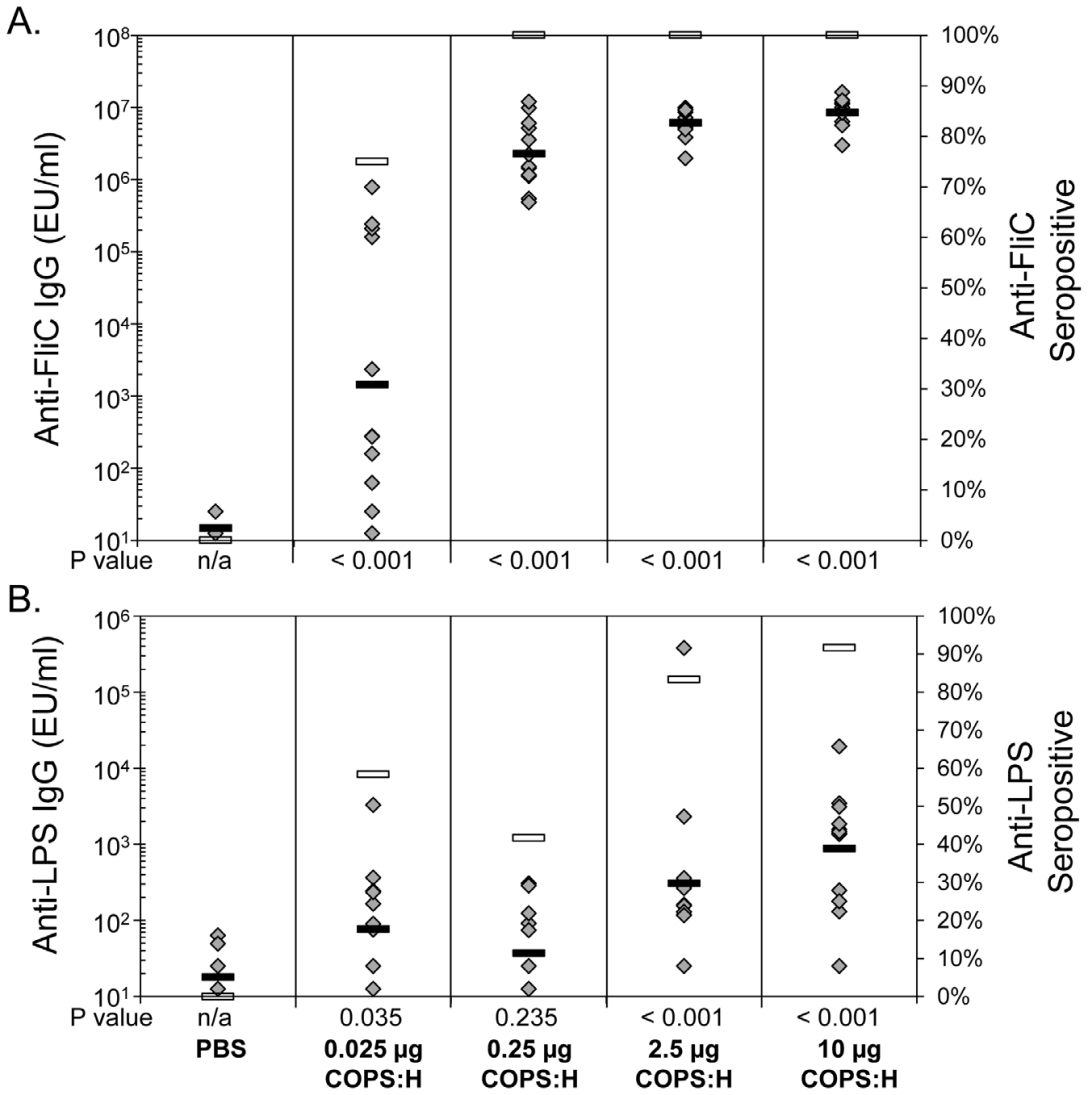
Serum titers and seropositive levels in mice receiving varying doses of COPS:FliC. Mice were immunized with PBS or three doses of the indicated amount of COPS:FliC. Serum was obtained 21 days after the third dose, and levels of specific antibody in individual mice (grey diamonds), and the geometric mean titer (black rectangles) and percent seropositive (white rectangles) within groups were determined by ELISA for A. FliC, or B. LPS. Statistical significance (P values) for COPS:FliC immunized groups relative to PBS controls assessed by Mann-Whitney rank sum test are indicated.

Higher geometric mean titers (GMT) and seropositive levels against both antigens were seen as a function of immunization with increasing amounts of COPS:FliC. Maximal titers (GMT = 2,000,000–6,000,000 EU/ml) and minimal animal-to-animal variation (100% seropositive) for anti-FliC IgG were achieved at dosages ≥0.25 µg (Fig. 1A). Mice immunized with 0.025 µg COPS:FliC (a 1/100^th^ fractional dose), also exhibited elevated anti-FliC IgG compared to controls, but the titers were lower (GMT = 1,449 EU/ml) and in some mice no anti-FliC IgG antibody responses could be detected (only 75% of mice were seropositive post-immunization). Anti-LPS IgG titers were generally lower and with higher animal-to-animal variability compared to anti-FliC IgG titers (Figure 1). Immunization with 10 µg (4-fold dose) or 2.5 µg (a full dose) of COPS:FliC elicited anti-LPS IgG GMTs of 885 EU/ml and 308 EU/ml, respectively, whereas immunization with 0.25 µg (1/10^th^ fractional dose) or 0.025 µg (1/100^th^ fractional dose) resulted in GMT’s of <80 EU/ml. Anti-LPS IgG was detected in 92% of the mice immunized with the 10 µg dosage level and in 83% of mice given a 2.5 µg dose. However, less than 60% of animals became seropositive for anti-LPS IgG after immunization with 0.25 µg or 0.025 µg of COPS:FliC.

### Protection after Immunization

IP challenge with wild type strain R11 caused 100% mortality in controls (Table 1). Mice actively immunized with three doses of COPS:FliC were significantly protected at all dosage levels tested (≥90% vaccine efficacy).

**Table 1:** Efficacy of different doses of *Salmonella* Enteritidis COPS:FliC conjugate in protecting mice from lethal challenge with wild-type *S.* Enteritidis R11^a^.

Vaccine	Dose	Mortality (dead/total)	Vaccine efficacy
PBS	−	12/12	−
COPS:FliC	0.025 µg	1/12^b^	90%
COPS:FliC	0.25 µg	0/12^b^	100%
COPS:FliC	2.5 µg	0/12^b^	100%
COPS:FliC	10 µg	0/12^b^	100%

a Mice challenged by the intraperitoneal route with 1×10^6^ CFU.

b p<0.001 compared to PBS control animals by two-tailed Fisher’s exact test.

### Protection by Passive Immunization

Challenge with R11 caused >80% mortality in mice to whom normal serum or PBS was passively administered (Table 2). In contrast, mice to whom immune sera from COPS:FliC conjugate-immunized mice was passively transferred were significantly protected from fatal *S*. Enteritidis challenge, as <15% mortality was observed (p = 0.005 versus normal serum controls).

**Table 2:** Efficacy of passive immunization into naïve mice with sera from mice immunized with COPS:FliC in protecting mice from lethal challenge with wild-type *S.* Enteritidis R11^a^.

Treatment	Mortality (dead/total)
PBS	5/6
Normal serum	7/7
COPS:FliC serum	1/7^b^

a Mice challenged by the intraperitoneal route with 5×10^5^ CFU.

b p = 0.005 compared to mice receiving normal serum by two-tailed Fisher’s exact test.

## Discussion

Vaccine strategies focused towards generating antibodies against *Salmonella* Typhi capsular polysaccharide are effective in preventing typhoid fever in humans. Unconjugated Vi polysaccharide vaccines are licensed for use in adults and older children and provide ∼55–60% protection for up to three years [9], before antibody levels plummet. A Vi-conjugate vaccine consisting of Vi linked to recombinant exoprotein A (rEPA) of *Pseudomonas aeruginosa* was immunogenic in Vietnamese children, pre-school children, toddlers and infants [10], [11], [12]. In a large-scale, randomized, controlled field trial in pre-school children in Vietnam, the Vi-rEPA conjugate exhibited 89% efficacy over 46 months of follow-up [10], [13]. Following the pioneering path blazed by Vi-rEPA conjugate in preventing typhoid fever, COPS-carrier protein glycoconjugate vaccines are now being pursued as a strategy to prevent paratyphoid A fever and invasive NTS infections [14], [15], [16].

Parenteral conjugate vaccines evoke primarily humoral systemic immune responses. Although *Salmonella* are intracellular pathogens, they are vulnerable to antibodies while they are extracellular during bacteremic dissemination [17]. Various critical threshold levels of serum IgG to Vi have been proposed as a correlate of the protection elicited by Vi-based vaccines [9], [18], [19]. Nevertheless, the antibody mediators and mechanistic correlates of protection in humans against NTS are as yet undefined. Accumulating evidence indicates that anti-*Salmonella* antibodies function through two main (and measurable) mechanisms, direct serum bactericidal activity (SBA) via the C9 complement membrane attack complex, and opsonophagocytosis of bacteria into phagocytes.

The importance of SBA to NTS is unclear as isolates from blood of certain serovars are resistant to complement-mediated lysis through the expression of long-chain OPS and the protein encoded by the resistance to complement killing (*rck*) gene [20], [21]. Nevertheless, both complement-resistant and susceptible *S*. Typhimurium strains are similarly susceptible to opsonophagocytic uptake and killing by oxidative burst [4], [22], [23]. We reported opsonophagocytic activity in sera from mice immunized with COPS:FliC [4]. That passively transferred cell-free immune sera from mice immunized with the highest dose of COPS:FliC (10 µg) recapitulated the protection seen with active immunization indicates that protection is likely mediated by systemic antibodies.

Using the homologous FliC as the carrier protein for conjugation to COPS offers several advantages. Antibody titers to the FliC carrier protein were generally higher than those to COPS. Protection at lower vaccine dosage levels could be due to high antibody levels to FliC, or to modest antibody responses to both antigens if the biological activities of anti-COPS and anti-FliC work synergistically. If in future clinical trials *Salmonella* COPS:FliC glycoconjugates prove to be protective vaccines in humans, these possibilities should be considered in attempting to identify antibody titer cut-offs that constitute a threshold for protective immunity.

Immune responses directed at the COPS hapten and to a carrier protein representing a protective antigen of the homologous pathogen could function synergistically to limit immune escape. The possible selection of Vi-negative *S*. Typhi strains has been raised as constituting a potential theoretical consequence if Vi-based vaccines were to become widely used in populations in endemic areas [24], [25]. In some endemic areas, *S*. Typhi putatively lacking Vi have in rare instances been isolated from the blood [26], [27]. However, it is presumed that susceptibility to complement exerts selective pressure similarly for both *S*. Typhi Vi and NTS long-chain OPS expression [20], [28]. Monophasic variants of *S*. Typhimurium lacking phase 2 flagellin FljB have been reported [29]. Isolates presumed devoid of flagella (i.e., non-motile, H- strains) apparently derived from *S*. Typhimurium parents occur but are rare [29]. *S*. Typhimurium mutants deficient in flagellin are pathogenic after oral infection in mice, however expression of flagella is documented as an important virulence determinant that contributes to cell invasion and inflammation *in vitro* [30]. Most circulating *S*. Typhimurium, *S*. Enteritidis and other serovar NTS strains associated with invasive disease are expected to be vulnerable to both anti-FliC and anti-OPS antibodies. The primary results reported herein demonstrate the efficacy of small fractional doses of *S*. Enteritidis COPS:FliC conjugate and document that passively transferred antibodies confer protection. These observations provide further impetus for pursuing this conjugate vaccine strategy to control invasive NTS disease in young children in sub-Saharan Africa.
